# Magnetic resonance imaging of regional gray matter volume in persons who died by suicide

**DOI:** 10.1038/s41380-024-02730-2

**Published:** 2024-09-05

**Authors:** E. Deininger-Czermak, L. Spencer, N. Zoelch, A. Sankar, D. Gascho, R. Guggenberger, S. Mathieu, M. J. Thali, H. P. Blumberg

**Affiliations:** 1 Department of Forensic Medicine and Imaging, Institute of Forensic Medicine, University of Zurich, Zurich, Switzerland; 2 Department of Nuclear Medicine, University Hospital Zurich, University of Zurich, Zurich, Switzerland; 3 Departments of Psychiatry, Yale School of Medicine, New Haven, CT USA; 4 Department of Psychiatry, Psychotherapy and Psychosomatics, Hospital of Psychiatry, University of Zurich, Zurich, Switzerland; 5 Neurobiology Research Unit, Copenhagen University Hospital Rigshospitalet, Copenhagen, Denmark; 6 Institute of Diagnostic and Interventional Radiology, University Hospital Zurich, University of Zurich, Zurich, Switzerland

**Keywords:** Psychiatric disorders, Neuroscience

## Abstract

In vivo neuroimaging research in suicide attempters has shown alterations in frontal system brain regions subserving emotional regulation, motivation, and self-perception; however, data from living individuals is limited in clarifying risk for suicide death. Postmortem neuroimaging provides an approach to study the brain in persons who died by suicide. Here, whole brain voxel-based analyses of magnetic resonance imaging gray matter volume measures were performed comparing persons confirmed by forensic investigation to have died by suicide (n = 24), versus other causes (n = 24), in a univariate model covarying for age and total brain volume; all subjects were scanned within 24 hours after death. Consistent with the hypothesis that persons who died by suicide would show lower gray matter volume in frontal system brain regions, this study of suicides showed lower gray matter volume in ventral frontal and its major connection sites including insula, striatum, and amygdala.

## Introduction

Suicide accounts for the death of about 800,000 people every year globally [1]. The neurobiology of risk for suicide has been investigated in the past few decades using neuroimaging, primarily by studying individuals with suicidal thoughts and behaviors (STBs) [2]. Those antemortem studies showed alterations in a frontal brain system that subserves emotional and reward-related processes, impulse control, decision-making, self-reflection, and pain and other interoceptive experiences, that include ventral prefrontal cortex (VPFC), anterior cingulate cortex (ACC), and insula, and their subcortical striatal and mesial temporal and posterior projection regions [2, 3]. However, antemortem studies are limited in informing on brain structures involved in the risk for death by suicide, since the individuals may not actually commit suicide.

Postmortem brain studies, although there are in number much less than in-vivo studies, have been also conducted to investigate structural alterations in suicide completers [4]. Compared to in-vivo studies, most postmortem studies have been of tissue samples and focused on cellular and molecular abnormalities, e.g. showing lower neuron density, and alterations in serotonin transporter binding, immunological factors, and gene expression, that have informed the field about potential mechanisms. The findings were often concentrated in the VPFC in suicides supporting its suggested role in suicides [5, 6]. Although rare MRI studies have been conducted postmortem, mainly in the context of Alzheimer disease or mood and psychotic disorders, postmortem imaging was shown to be conducted successfully [7, 8]. The main challenges in postmortem MRI are decomposition processes, including gas formation and loss of white and grey matter differentiation. Therefore, time is an essential factor in scanning postmortem subjects. Studies on fixed tissues of deceased persons are additionally limited by potential shrinkage of the tissue due to fixation. Here, we compared structural magnetic resonance imaging (MRI) data obtained from scanning a group with forensically clarified suicide (n = 24) to a group who died by natural causes (n = 24), within 24 hours after their estimated time of death (demographic, clinical and cause of death details are provided in Table 1). We hypothesized that persons who died by suicide would show lower gray matter volume in the frontal system, based on the previously published in-vivo results [2]. As this was a rare MRI study of suicides, it was considered important not to have premature closure on involved brain areas. Therefore, voxel-based morphometry was used to assess the whole brain.

**Table 1:** Details of all suicide cases including age, sex, suicide method, previous suicidal ideation or behavior, known psychiatric disorder and drugs detected at toxicologic screening.

Case	Sex	Age	Suicide Method	Previous Suicidal Ideation or Behavior	Diagnosed Psychiatric Disorder	Toxicologic Screening
1	f	22	Hanging	Attempt	Yes*	
2	m	31	Hanging	Ideation		SNRI, alcohol
3	m	32	Hanging	Attempt		Alcohol, cocaine
4	m	33	Hanging	Attempt	Bipolar disorder	Cannabis, SSRI
5	m	49	Hanging	Ideation		Alcohol, benzodiazepine, atypical antipsychotic, SNRI
6	m	50	Hanging	Unknown	Psychotic disorder	Atypical antipsychotic, SNRI, atypical antipsychotic, benzodiazepine
7	m	53	Hanging	Unknown		
8	m	55	Hanging	Unknown	Schizophrenia	Benzodiazepine
9	m	20	Hanging	Ideation		
10	m	18	Intoxication	Attempt	Autism Spectrum Disorder	Potassium cyanide
11	m	30	Intoxication	Unknown	Yes*	Benzodiazepine, SNRI
12	m	33	Intoxication	None		(Iso-)Taxin B, Aconitine
13	m	44	Intoxication	Unknown	Depression	SNRI, atypical antidepressant
14	f	52	Intoxication	Attempt	Panic attacks	Benzodiazepine, SSRI
15	m	73	Intoxication	Ideation		Barbiturate
16	f	77	Intoxication	Ideation		Barbiturate
17	m	82	Intoxication	Unknown		Benzodiazepine
18	m	90	Intoxication	Ideation		Barbiturate
19	f	86	Firearm	Ideation		
20	m	90	Firearm	Ideation		
21	m	93	Firearm	Ideation		
22	m	37	CO	Ideation		
23	m	57	CO	None		
24	m	34	Helium			

Toxicologic screening included blood and/or urine samples.

*CO* Carbon Monoxide, *f* Female, *m* Male, *SNRI* Serotonin and Norepinephrine Reuptake Inhibitors, *SSRI* Selective Serotonin Reuptake Inhibitor.

*A psychiatric disorder was confirmed and stated in forensic reports, however, no specific diagnosis was documented.

## Material and methods

### Subjects

Subjects included 48 adult decedents (24 suicide cases; 24 controls). The mean age ± standard deviation in the suicide group was 51 ± 24 years and in the control group 51 ± 18 years. The two groups had an identical gender distribution of males (n = 20) and females (n = 4) each and did not differ significantly in body mass index (suicide group 23.1 ± 4.3, control group 27.61 ± 6.7). The cause and manner of death was confirmed by forensic pathologists. Only subjects who underwent CT and MRI scans within a maximum of 24 hours after the estimated time of death were considered for this study to minimize potential bias caused by postmortem changes such as intraparenchymal gas formation. In all cases, the temperature was above 10°C (minimum=11°C, maximum=35°C, mean=20°C), determined using magnetic resonance spectroscopy thermometry which was measured intracranially in the supraventricular white matter, as low temperatures have been reported to influence T1 contrast [9].

In the control group, a non-natural manner of death was forensically excluded; the leading cause was cardiovascular events (79.2%; 19/24). In 1 case (4.2%), an infection was named as cause of death. In 4 cases (16.7%), the cause remained unknown after autopsy, but the manner of death was deemed clearly natural.

In the suicide group, the manner of death was classified as non-natural death by suicide: 50% committed suicide through a mechanical method (hanging n = 8 or gunshots n = 3), and 50% through a non-mechanical method (drug intoxication n = 9 or inhalation of carbon-monoxide n = 2 or helium n = 1). Based on police reports and interviews of the decedent’s next of kin, 58.3% (n = 14) of the subjects in the suicide group were known to have had suicidal ideation (n = 9) or attempted suicide previously (n = 5); in 41.7% (n = 10), previous suicidal behavior could not be evaluated by the investigators, e.g., because the next of kin did not declare any or it was not considered essential for the case. Psychiatric disorders were diagnosed in 33.3% (n = 8) of the suicide group, which were documented in their medical charts. In 37.5% (n = 9), medical history was retrievable, however, the presence of a psychiatric disorder could not be conclusively determined, either because of vague descriptions of possible disorders or incomplete medical histories. In the remaining 29.2% (n = 7) cases of the suicide group, investigators did not identify data to conclusively support any psychiatric disorders; however, medical histories were not completely retrievable. None of the cases in the control group had conclusive evidence of a psychiatric disorder. In all suicide cases, a postmortem toxicologic screening was conducted, whereas in the control group, this was only conducted, if possible drug use antemortem was suspected by the investigators (n = 8), which showed alcohol consumption prior to death in only one case. Toxicologic testing in the suicide cohort confirmed use of psychotropic medications; this included use of only one medication class (selective serotonin reuptake inhibitors (SSRI; n = 3), benzodiazepines (n = 2) or barbiturates (n = 3)) alone, or a combination of different medication classes, including benzodiazepines with SSRIs (n = 2), SSRIs with antipsychotics and benzodiazepines (n = 2) and SSRIs with antipsychotics (n = 1). In one case, cocaine abuse was confirmed by toxicology. Four subjects in the suicide group consumed alcohol before death. Subject details are listed in Table 1.

Exclusion criteria for both groups included traumatic injuries and surgeries to the head/brain. The control group was also without psychiatric disorders, positive toxicologic screen for psychotropic medication, or suspected drug usage. All MRI images were also visually inspected by the same neuroradiologist to confirm that there were no visible cerebral lesions or intracranial masses. Corticomedullar differentiation had to be normal on MRI to exclude pathology and relevant postmortem changes. Subjects were also excluded for postmortem-related changes such as intraparenchymal gas or parenchymal volume loss due to decomposition.

### Imaging acquisition

#### CT

A 128-multislice-detector CT scanner (Somatom Definition Flash, Siemens Healthineers, Forchheim, Germany) was used for all CT scans. The scan protocol was adapted according to postmortem scan recommendations using a tube voltage of 120 kVp, tube current of 1000 mAs. No dose modulation was applied [10]. The image dataset was reconstructed transaxially at a slice thickness of 0.6 mm and an increment of 0.4 mm using a bone kernel (H60) and a soft kernel (H31). A maximum field of view of 300 × 300 mm^2^ with a matrix of 512 × 512 was used.

#### MRI

All subjects were scanned with a 3-Tesla MRI scanner (Achieva 3.0 TX, Philips Healthcare, Best, the Netherlands) and an 8-channel phased-array receive-only head coil (Philips Healthcare, Best, NL). In the scan protocol, a three-dimensional T1-weighted sequence (inversion recovery turbo field echo sequence) was included using parameters: repetition time 9.4 ms, echo time 4.6 ms, inversion time 600 ms, flip angle 8°, field of view: 240 mm x 240 mm x 182 mm and 1 mm isotropic voxel resolution.

### Analyses

For evaluation of brain images, Syngo.via (Version 30A_HF91, Siemens, Healthcare) was used. To exclude bone lesions, including fractures, all CT images were visually inspected by a board-certified neuroradiologist with more than 10 years of experience (R.G.), using images reconstructed with a standard kernel for bone (B60).

#### Neuroimaging analyses

All acquired images were processed using MATLAB R2021b (MathWorks, Sherborn, MA) and Statistical Parametric Mapping (SPM12) [11]. The SPM segmentation function and SPM tissue probability maps for gray matter, white matter, and cerebral spinal fluid were used for bias correction and segmentation. Data were normalized to Montreal Neurological Institute (MNI) space and smoothed with an 8-mm full‐width‐at‐half‐maximum isotropic kernel.

A two-sample *t* test in SPM12 was used to evaluate voxel-based group differences in grey matter volume (GMV) throughout the brain. Covariates included age, to minimize the potential confounds of a range of ages of the subjects, and total brain volume to control for inter-individual variations in brain size. For this primary analysis, clusters were considered significant at a voxel wise *p* < 0.005, and if they survived a cluster-level threshold corrected for multiple comparisons (Family-Wise Error (FWE) correction) of *p*_*fwe*_ < 0.05. Clusters only surviving the voxel-level threshold of *p* < 0.005 with a cluster threshold of 20 voxels (*p*_*uncorrected*_ < 0.005) were considered trend-level findings and reported given the rarity of such study.

From clusters showing a significant or trending difference between the control and suicide groups, mean GMV values were extracted with the MarsBaR toolbox (http://marsbar.sourceforge.net) and used as dependent variables in post hoc analysis using SPSS version 29.0 (IBM, Armonk, New York) to explore for potential differences between persons who died by suicide in whether they died by intoxication versus mechanical methods.

## Results

**Fig. 1:**
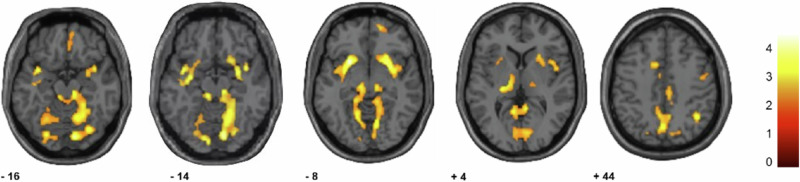
Regions of decreased gray matter volume in persons who died by suicide compared to persons who died by other methods. The axial-oblique slices show clusters of lower gray matter volume in the suicide group at *p* < 0.005, uncorrected. The right hemisphere is shown on the right side of the figure, the numbers under the images are the mm in the z-plane for the slice in Montreal Neurological Institute (MNI) Atlas, and the color bar shows the range of z-score values. All clusters survived the significance threshold set to *p* < 0.05 family wise error correction with the exception of the right orbital and rostromedial prefrontal cortex regions (anterior-most cluster shown on slices z = −16mm and -8mm) that showed a trend towards significance (voxel-level threshold of *p* < 0.005 with a cluster threshold of 20 voxels). Regions of significantly decreased gray matter volume included ventral anterior cingulate cortex (VACC) (bilateral Brodman Area, BA, 25), left OFC (BA 47) and bilateral insula, temporopolar cortex (BA 38), parahippocampal gyrus, thalamus, ventral striatum, amygdala, as well as bilateral posterior cingulate cortex (BA 31), precuneus (BA 7), temporal and occipital association areas (BAs 18,19), brainstem and cerebellum, and right parietal cortex (BA 40).

In persons who died by suicide, significantly lower gray matter volume was observed (Fig. 1) in ventral frontal regions that included bilateral ventral anterior cingulate (VACC) (Brodmann Area, BA, 25) and left orbital PFC (BA 47) *p*_*fwe*_ < 0.05; trends were observed in right orbital and rostromedial PFC (BAs 11 and 10) *p*_*uncorrected*_ < 0.005 with a cluster size of 486 voxels. Significantly lower gray matter volume in suicides was also observed in bilateral insula, temporopolar cortex (BA 38), parahippocampal gyrus, thalamus, ventral striatum, amygdala, as well as bilateral posterior cingulate cortex (BA 31), precuneus (BA 7), temporal and occipital association areas (BAs 18,19), brainstem and cerebellum, and right parietal cortex (BA 40) *p*_*fwe*_ < 0.05. No significantly larger volumes were observed in the suicide group, and findings did not differ significantly by suicide method, i.e., lethal intoxication compared to mechanical methods (*p* > 0.35).

## Discussion

The findings in this study comparing MRI of individuals who died by suicide with individuals who died by other causes, scanned by MRI within 24 hours of their death, are consistent with convergent data from the study of individuals with STBs from over two decades that implicate ventral frontal regions and their major projection regions within a frontal brain system that subserves behavioral domains involved in suicide risk including emotional and other behavioral regulation, reward, self-perception, and pain [2]. These novel data support the involvement of these brain regions in the risk of death by suicide.

Gray matter volume reductions in persons who died by suicide were significant in orbital PFC and VACC ventral frontal regions that regions play prominent roles in depression and other major behavior risk factors for suicide including hopelessness, making them a target for neuroanatomical treatments for depression such as deep brain stimulation and for measuring treatment response [12, 13]. Together with the current findings, the prior work implicates them as targets for suicide prevention, supported by their changes associated with rapid suicidal ideation reductions with ketamine [14].

The ventral frontal regions have strong projections to the insula and temporopolar cortical regions, together with them comprising anterior paralimbic cortices with important roles in emotions, as well as additional behavioral domains highly implicated in STBs [15, 16]. For example, these anterior paralimbic cortices have strong connectivity with the hypothalamus which subserves functions also associated with suicide including stress responses and sleep disturbances [17, 18]. Insula involvement is especially of interest given its role in self-awareness, pain, and other interoceptive processes and guidance of social behavior implicated in STBs, and has been studied as a potential biomarker of mood treatment response [19, 20]. These anterior paralimbic regions also have strong connections to subcortical regions, including the amygdala important in emotion and the ventral striatum in motivational and reward processes; anhedonia is a known suicide risk factor and is also a target for treatment-resistant depression [21, 22]. Other regions in which findings were observed and that were previously associated to STBs included parahippocampal regions implicated in memory, and posterior association regions, which play important roles in external perception [23, 24]. While the processes subserved by these regions are consistent with the ones involved in suicide risk, we cannot conclude from the data of this study the functional consequences of the morphological differences.

The postmortem MRI findings of this study are also consistent with prior postmortem studies of fixed tissue of persons who died by suicide. These studies, informative in identifying cellular and molecular involvement, for example, have found VPFC differences serotonergic system-related differences [5, 25]. Differences were not observed in volumes of significant clusters between suicide cases with versus without positive drug screens; however, screens were performed after death, and information such as the duration of drug use also relied on available medical charts and next of kin information. It also cannot be determined whether findings might be related to brain changes due to the suicide method. These could lead to findings such as brain edema for example due to drug use, or toxic inhalation, although significant gray matter differences were not observed in use of lethal intoxication compared to mechanical methods. Although the bias of postmortem brain changes such as edema cannot be completely excluded, a neuroradiologist assessed all images in regard to corticomedullary differentiation, brain herniation, and ventricle width to reduce this possible influence, and analyses were performed covarying for total brain volume. The postmortem study types share limitations. Sample sizes are modest, which limits the ability to assess for the potential influences of subject heterogeneity. It is difficult to determine whether the findings are related to suicide risk or psychopathology that may have contributed to it and rely on prior history documents and next of kin reports that may be incomplete. Further, ability to attribute findings specifically to suicide are limited without a control group of individuals who died by suicide but who were confirmed to not have psychopathology. Thus, it is possible that findings may be at least partially related to psychopathology. It was also not possible to reliably ascertain comprehensive tobacco smoking histories, which is another potential confound.

In conclusion, we observed decreased volumes in the suicide group in frontal system brain regions consistent with previous antemortem neuroimaging and behavioral studies of suicide thoughts and behaviors, and postmortem fixed tissue studies, extending those results to support the involvement of those brain regions in suicide and as targets for suicide prevention.

## Data Availability

The datasets generated and analyzed during the current study are not publicly available due to reasons of data privacy and regulatory/ethical approvals, but aggregate-level data are available from the corresponding author upon reasonable request and pending local and national data protection and ethics regulations.

## References

[1] Organization WH. World health statistics overview 2019: monitoring health for the SDGs, sustainable development goals: World Health Organization; 2019.

[2] Schmaal L, van Harmelen A-L, Chatzi V, Lippard ET, Toenders YJ, Averill LA, et al. Imaging suicidal thoughts and behaviors: a comprehensive review of 2 decades of neuroimaging studies. Mol psychiatry. 2020;25:408–27. 31787757 10.1038/s41380-019-0587-x PMC6974434

[3] Chen C-f, Chen W-n, Zhang B. Functional alterations of the suicidal brain: a coordinate-based meta-analysis of functional imaging studies. Brain Imaging Behav. 2022;16:291–304. 34351557 10.1007/s11682-021-00503-x

[4] Arango V, Underwood MD, Mann JJ. Postmortem findings in suicide victims. Implications for in vivo imaging studies. Ann N. Y Acad Sci. 1997;836:269–87. 9616804 10.1111/j.1749-6632.1997.tb52365.x

[5] Mann JJ, Brent DA, Arango V. The neurobiology and genetics of suicide and attempted suicide:: a focus on the serotonergic system. Neuropsychopharmacology. 2001;24:467–77. 11282247 10.1016/S0893-133X(00)00228-1

[6] García-Gutiérrez MS, Navarrete F, Navarro G, Reyes-Resina I, Franco R, Lanciego JL, et al. Alterations in gene and protein expression of cannabinoid CB 2 and GPR55 receptors in the dorsolateral prefrontal cortex of suicide victims. Neurotherapeutics. 2018;15:796–806. 29435814 10.1007/s13311-018-0610-y PMC6095782

[7] Bobinski M, De Leon M, Wegiel J, Desanti S, Convit A, Saint Louis L, et al. The histological validation of post mortem magnetic resonance imaging-determined hippocampal volume in Alzheimer’s disease. Neuroscience. 1999;95:721–5. 10.1016/s0306-4522(99)00476-5 10670438

[8] Busch J, Lundemose S, Lynnerup N, Jacobsen C, Jørgensen M, Banner J. Post-mortem MRI-based volumetry of the hippocampus in forensic cases of decedents with severe mental illness. Forensic Sci, Med Pathol. 2019;15:213–7. 30828766 10.1007/s12024-019-00101-w

[9] Zoelch N, Heimer J, Richter H, Luechinger R, Archibald J, Thali MJ, et al. In situ temperature determination using magnetic resonance spectroscopy thermometry for noninvasive postmortem examinations. 2024**:** e5171. 10.1002/nbm.5171 38757603

[10] Gascho D, Thali MJ, Niemann T. Post-mortem computed tomography: technical principles and recommended parameter settings for high-resolution imaging. Med, Sci Law. 2018;58:70–82. 29310502 10.1177/0025802417747167

[11] Ashburner J, Barnes G, Chen C-C, Daunizeau J, Flandin G, Friston K, et al. SPM12 manual. *Wellcome Trust Centre for Neuroimaging, London, UK* 2014;2464.

[12] Sankar A, Purves K, Colic L, Cox Lippard ET, Millard H, Fan S, et al. Altered frontal cortex functioning in emotion regulation and hopelessness in bipolar disorder. Bipolar Disord. 2021;23:152–64. 32521570 10.1111/bdi.12954 PMC7790437

[13] Holtzheimer PE, Husain MM, Lisanby SH, Taylor SF, Whitworth LA, McClintock S, et al. Subcallosal cingulate deep brain stimulation for treatment-resistant depression: a multisite, randomised, sham-controlled trial. Lancet Psychiatry. 2017;4:839–49. 28988904 10.1016/S2215-0366(17)30371-1

[14] Ballard ED, Lally N, Nugent AC, Furey ML, Luckenbaugh DA, Zarate CA Jr. Neural correlates of suicidal ideation and its reduction in depression. Int J Neuropsychopharmacol. 2015;18:pyu069. 10.1093/ijnp/pyu069 PMC4307932 25550331

[15] Morecraft R, Geula C, Mesulam MM. Cytoarchitecture and neural afferents of orbitofrontal cortex in the brain of the monkey. J Comp Neurol. 1992;323:341–58. 1460107 10.1002/cne.903230304

[16] Sankar A, Scheinost D, Goldman DA, Drachman R, Colic L, Villa LM, et al. Graph theory analysis of whole brain functional connectivity to assess disturbances associated with suicide attempts in bipolar disorder. Transl psychiatry. 2022;12:7. 35013103 10.1038/s41398-021-01767-z PMC8748935

[17] Porras-Segovia A, Perez-Rodriguez MM, López-Esteban P, Courtet P, López-Castromán J, Cervilla JA, et al. Contribution of sleep deprivation to suicidal behaviour: a systematic review. Sleep Med Rev. 2019;44:37–47. 30640161 10.1016/j.smrv.2018.12.005

[18] O’Connor DB, Gartland N, O’Connor RC. Stress, cortisol and suicide risk. Int Rev Neurobiol. 2020;152:101–30. 32450993 10.1016/bs.irn.2019.11.006

[19] Benarroch EE. Insular cortex: functional complexity and clinical correlations. Neurology. 2019;93:932–8. 31645470 10.1212/WNL.0000000000008525

[20] Kelley ME, Choi KS, Rajendra JK, Craighead WE, Rakofsky JJ, Dunlop BW, et al. Establishing evidence for clinical utility of a neuroimaging biomarker in major depressive disorder: prospective testing and implementation challenges. Biol Psychiatry. 2021;90:236–42. 33896622 10.1016/j.biopsych.2021.02.966 PMC8324510

[21] Auerbach RP, Pisoni A, Bondy E, Kumar P, Stewart JG, Yendiki A, et al. Neuroanatomical prediction of anhedonia in adolescents. Neuropsychopharmacology. 2017;42:2087–95. 28165037 10.1038/npp.2017.28 PMC5561341

[22] Pizzagalli DA. Toward a better understanding of the mechanisms and pathophysiology of Anhedonia: Are we ready for translation? Am J Psychiatry. 2022;179:458–69. 35775159 10.1176/appi.ajp.20220423 PMC9308971

[23] Wei S, Chang M, Zhang R, Jiang X, Wang F, Tang Y. Amygdala functional connectivity in female patients with major depressive disorder with and without suicidal ideation. Ann Gen Psychiatry. 2018;17:1–7. 10.1186/s12991-018-0208-0 PMC6134510 30214465

[24] Zhang S, Chen J-m, Kuang L, Cao J, Zhang H, Ai M, et al. Association between abnormal default mode network activity and suicidality in depressed adolescents. BMC psychiatry. 2016;16:1–10. 27688124 10.1186/s12888-016-1047-7 PMC5041526

[25] Pandey GN, Dwivedi Y, Rizavi HS, Ren X, Pandey SC, Pesold C, et al. Higher expression of serotonin 5-HT2A receptors in the postmortem brains of teenage suicide victims. Am J Psychiatry. 2002;159:419–29. 11870006 10.1176/appi.ajp.159.3.419

